# Opportunities and challenges for delivering non-communicable disease management and services in fragile and post-conflict settings: perceptions of policy-makers and health providers in Sierra Leone

**DOI:** 10.1186/s13031-019-0248-3

**Published:** 2020-01-06

**Authors:** Sophie Witter, Guanyang Zou, Karin Diaconu, Reynold G. B. Senesi, Ayesha Idriss, John Walley, Haja Ramatulai Wurie

**Affiliations:** 1 Institute for Global Health and Development, Queen Margaret University, Edinburgh, UK; 2 School of Economics and Management, Guangzhou University of Chinese Medicine, Guangzhou, China; 3 Directorate of Non-Communicable Diseases and Mental Health, Ministry of Health and Sanitation of Sierra Leone, Freetown, Sierra Leone; 4 Nuffield Centre for International Health and Development, University of Leeds, Leeds, UK; 5 College of Medicine and Allied Health Sciences, University of Sierra Leone, Freetown, Sierra Leone

**Keywords:** Non-communicable disease, Health systems assessment, Fragile and post-conflict settings, Sierra Leone

## Abstract

**Background:**

The growing burden of non-communicable diseases in low- and middle-income countries presents substantive challenges for health systems. This is also the case in fragile, post-conflict and post-Ebola Sierra Leone, where NCDs represent an increasingly significant disease burden (around 30% of adult men and women have raised blood pressure). To date, documentation of health system challenges and opportunities for NCD prevention and control is limited in such settings. This paper aims to identify opportunities and challenges in provision of NCD prevention and care and highlight lessons for Sierra Leone and other fragile states in the battle against the growing NCD epidemic.

**Methods:**

This paper focuses on the case of Sierra Leone and uses a combination of participatory group model building at national and district level, in rural and urban districts, interviews with 28 key informants and review of secondary data and documents. Data is analysed using the WHO’s health system assessment guide for NCDs.

**Results:**

We highlight multiple challenges typical to those encountered in other fragile settings to the delivery of preventive and curative NCD services. There is limited government and donor commitment to financing and implementation of the national NCD policy and strategy, limited and poorly distributed health workforce and pharmaceuticals, high financial barriers for users, and lack of access to quality-assured medicines with consequent high recourse to private and informal care seeking. We identify how to strengthen the system within existing (low) resources, including through improved clinical guides and tools, more effective engagement with communities, and regulatory and fiscal measures.

**Conclusion:**

Our study suggests that NCD prevention and control is of low but increasing priority in Sierra Leone; challenges to addressing this burden relate to huge numbers with NCDs (especially hypertension) requiring care, overall resource constraints and wider systemic issues, including poorly supported primary care services and access barriers. In addition to securing and strengthening political will and commitment and directing more resources and attention towards this area, there is a need for in-depth exploratory and implementation research to shape and test NCD interventions in fragile and post-conflict settings.

## Introduction

Non-communicable diseases (NCDs), mainly hypertension, diabetes, cardiovascular disease, cancer, and chronic respiratory diseases, kill 41 million people each year, contributing to 71% of global deaths [1]. NCDs disproportionately affect people in low- and middle-income countries (LMICs), where more than 75% of global NCD deaths occur. The growing burden of NCDs in LMICs adds to existing health threats and worsens poverty. Such challenges are exacerbated in fragile settings, which are characterised by weak health systems where prevention and management are very challenging [2]. Health systems in many Sub-Saharan African countries remain fragile, fragmented, under-resourced and limited for mounting an effective response to the double burden of communicable diseases and NCDs [3].

Increasingly, fragility is recognized as a multi-dimensional phenomena reflective of both country and historical context, the vulnerabilities of the health system and population itself, as well as breakdowns in legitimizing interactions between populations and health systems [4]. Sierra Leone exhibits evidence of multiple such dimensions: not only is the country still attempting to stabilize and mature after a contested history and long-lasting and brutal civil war, the health system itself is recognized as one of the most fragile systems in the world and continues to struggle to rebuild despite epidemics and severe financing and human resource constraints [5]. Given these challenges, efforts were focused on communicable diseases and reproductive health post-conflict. However, the NCD burden has been growing. The World Health Organization (WHO) estimated that the percentage of deaths attributable to NCDs in Sierra Leone was 18% in 2008 and this has increased to 26% in 2012, with cardiovascular diseases accounting for 9% [6]. WHO further estimated that 30% of men and 31% of women over 18 years had raised blood pressure, while nearly 5% of adults had raised blood glucose in 2014 [6]. Other risk factors of NCDs are also common: 33% of men and 6% of women smoked every day, and 9 and 28% of adults were estimated to be obese and overweight respectively [6] .

The population in Sierra Leone is additionally recognized as one of the most vulnerable globally, including due to iniquitous health service delivery. Recent research suggests that despite widespread social mobilization in the wake of Ebola, trust in the health care system still presents a substantive problem to health service utilization [7, 8]. This is of critical importance to NCD prevention and management in particular, given the need for care continuity. Currently, NCDs are managed and treated at all levels of the health care system, informed by guidelines provided by the NCD and mental health directorate of the Ministry of Health and Sanitation before (MoHS). For example, hypertension can be managed at the primary health care level, with established referral pathways to ensure that severe complications from hypertension are referred from primary health care level to secondary or tertiary care facilities [9]. However, cardiovascular disease, diabetes and cancer are largely managed in tertiary or secondary-level hospitals. Patients however have to pay to access NCD services within government health facilities, with the exception of NCD services provided by Partners in Health (at Koidu Government Hospital).

To date, documentation of health system challenges and opportunities for NCD prevention and control is limited in LMICs, and especially in sub-Saharan Africa and fragile and post-conflict settings. Much of the literature on NCDs in sub-Saharan Africa focuses on documenting the prevalence of NCDs and their (multiple) risk factors [10–16] as well as providing a socioeconomic and gender breakdown for these [17, 18]. Increasingly, the comorbidity of NCDs and infectious chronic diseases (such as HIV/AIDS) [19, 20] has also been recognized. The literature analysing health systems challenges to NCD prevention and management in this particular region, and focusing on fragile settings, is still in its infancy however. Studies predominantly focus on particular aspects of the health system. These include, for example, gaps in staff training to manage NCDs [21]; incomplete NCD burden data [3], low knowledge and awareness of NCD burdens, drivers and impact, and non-availability of relatively inexpensive NCD medications in public sector facilities [22]; patients’ knowledge gaps regarding the preventable aspects of hypertension and diabetes and mistrust in the health care system [23]; limited progress on guidelines for management of NCD and drug therapy and counselling [24]; lack of multi-sectoral collaboration [25]; poor financial protection for NCD patients [26]; poor accessibility, equity and responsiveness of primary healthcare services for CVD [27]. However, few studies have conducted a comprehensive and systematic health systems assessment of NCD management and services in LMIC and fragile settings.

This paper aims to analyse opportunities and challenges in provision of NCD prevention and care in Sierra Leone, using mixed and participatory methods and drawing on the WHO assessment framework [28], in order to highlight lessons for it and other fragile states in the battle against the growing NCD epidemic.

## Methods

The paper takes a cross-sectional approach, integrating data from primary and secondary sources, including key informant interviews, group model building (GMB) and secondary literature. Findings are structured according to the WHO’s health system assessment guide for NCDs [28]. This delineates a five-step process. The guide was pilot-tested in 2013 in five countries - Hungary, Kyrgyzstan, Republic of Moldova, Tajikistan and Turkey and has since been used in other countries in the WHO EURO region, such as Armenia [29] and Estonia [30].

### Documentary review

A scoping review of published and grey studies on NCDs in Sierra Leone, including data on the burden of disease and risk factors, the policy context, health interventions and actors, was conducted in 2017 [31]. The literature was relatively limited: in total, 28 documents were included. Of these, 23 were published articles, four reports and one, a (MoHS) strategy [31]. Three reviewers extracted data according to a pre-defined template and employed narrative synthesis for analysis. In this article, we draw on this review, as well as relevant more recent documentary sources on NCDs in Sierra Leone.

### Key informant interviews and other stakeholder engagement activities

To further explore issues affecting service delivery and access to care at both micro- (clinic), meso- (district) and macro- (national) health system levels, 28 key informant interviews (18 male and 10 female) were conducted between April and September 2018, using a semi-structured interview guide (Additional file 1), in Western Area (urban) and Bombali district (rural).

Purposive and convenience sampling techniques were used to identify participants with experience relevant to NCD prevention and service delivery at each of the targeted system levels. Participants included national stakeholders, including in the Ministry of Health and Sanitation and tertiary institutions; development partners; District Health Management Team (DHMT) members, district hospital staff, community health officers (CHOs) and nursing staff at community health centres (CHCs), non-governmental organisations and technical partners at district level. Interviews took 30–45 min, and were either recorded or notes taken.

Informal interviews or meetings were also conducted with other stakeholders in Sierra Leone. For instance, the District Medical Officer in the rural district invited a group of health officers and CHOs to meet with us and discuss the NCD issues. These, together with the key informant interviews, informed scripts for the group model building.

Thematic analysis was used to analyse the qualitative data [32], following the WHO framework structure [28]. This data was triangulated with data from other sources under each domain by the team.

### Group model building

We conducted three participatory group model building sessions: one with national policy makers and district health representatives, one with urban health providers, and one with rural health care providers. We summarize the sessions conducted, participants involved and topics discussed in Table 1. Sessions were also held with communities but these are reported elsewhere [7].

**Table 1:** Overview of group model building sessions, Sierra Leone

Setting and date	Number of participants^a^			Profile of participants involved	Topics discussed
	Total	Male	Female		
National (Freetown) September 2018	21	12	9	Policy makers at national level; donors and WHO representatives, NGOs; district level health care professionals; technical experts and academics	Current policy and practice related challenges relating to NCD/MHPSS in Sierra Leone; Potential areas for intervention
Urban (Western Area) September 2018	20	10	10	Health care providers (i.e. doctors, nurses, CHOs,) involved in NCD/Mental Health and Psychosocial Support (MHPSS) service delivery in urban environments	Knowledge and perceptions of NCD/MHPSS conditions and their causes; Current practices and related challenges to NCD/MHPSS service delivery; Potential areas for intervention in the system.
Rural (Bombali district) June 2018	39	23	16	Health care providers (doctors, nurses, CHOs, community health workers) involved in NCD/MHPSS service delivery in rural environments – including local NGOs	Knowledge and perceptions of NCD/MHPSS conditions and their causes; Current practices and related challenges to NCD/MHPSS service delivery; sPotential areas for intervention in the system.
Total	80	56%	44%		

^a^This includes MHPSS participants

#### Participant sampling and recruitment

Participants were sampled purposively due the specific roles they performed within the health system. For example, in both urban and rural settings, we targeted health providers with experience in delivery of NCD services – i.e. doctors, nurses, clerks. We included both formal and informal care providers to capture a breadth of perspectives. Convenience and snow-ball sampling techniques were also employed. During each field visit, recruitment took place each time 1–2 days prior to the research team’s visit to a specific location. Research team members contacted local health care staff and local leaders and, based on their recommendations and mobilization, further contacted local members (either via phone or word of mouth) and invited them to the relevant group model building session.

#### Group model building sessions

Each group model building session was hosted in a location convenient to participants. The research team aimed to host sessions in locations separate to local health care facilities to minimize any potential discomfort for participants.

The research team started each group model building session by explaining the purpose of the project, informing participants that their presence and participation was voluntary and securing oral consent. Sessions lasted 4.5 h on average and activities were tailored to the specific topics to be discussed and group of participants involved. Each activity had a specific ‘script’ outlining the task and objectives at hand (Additional file 2). Scripts were elaborated and refined by the research group, drawing from publicly available scripts (Scriptopaedia) and research protocols on the study of social connections [33].

#### GMB analysis

Group model building sessions culminated in the development of incipient causal loop models which served as a basis for further analyses. Systems diagrams elaborated in group model building sessions were iteratively refined by the research team; analyses proceeded step-wise and additionally involved triangulation with further theoretical frameworks available. First, concepts/pictures drawn were clarified and as possible upon reflection of sessions, further pathways were added for completeness. Second, the research team identified the leverage points affecting the dynamics within each of the elaborated models. Third, models from different sites were compared, to identify salient differences due to setting specific characteristics.

## Results

Our results follow the WHO health system assessment framework for NCD provision (see Table 2).

**Table 2:** Common health system barriers and opportunities for NCD provision (WHO, 2015)

Developing political commitment	
Creating explicit processes for priority setting	
Strengthening interagency cooperation	
Enhancing population empowerment	
Establishing effective models of service delivery	
Establishing coordination across providers	
Taking advantage of economies of scale and specialisation	
Creating the right incentive systems	
Integrating evidence into planning	
Addressing human resources challenges	
Improving access to quality medicines for NCDs	
Strengthening health system management	
Creating adequate information solutions	
Overcoming resistance to change	
Ensuring access to care and reducing financial burdens	

### Developing political commitment

**Fig. 1:**
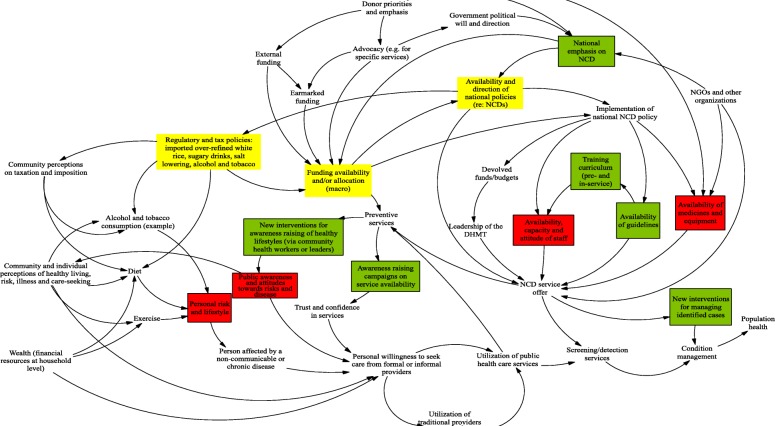
Causal loop diagram: national NCD stakeholders. Note: Red boxes mark points of fragility in the current NCD systems of care. Green boxes refer to intervention strategies participants elicited in response to fragility points – i.e. these are parts of the system that can be strengthened (largely) in the short-term. Yellow boxes refer to more fundamental parts of the NCD care system (NCD policy availability and direction, funding availability and financing mechanisms) which require substantive elaboration and debate prior to implementation

Sierra Leone’s national policies emphasise health system strengthening. However, past efforts have focused largely on reproductive, maternal and child health, as well as communicable diseases. Until recently, relatively limited attention has been paid to NCDs, which is reflected in the limited dedicated budget for NCD care [31]. Equally, international attention and funding has not been focused on this issue in contexts like Sierra Leone. The MoHS developed its first National NCD Policy [34] and National NCD Strategic Plan [35] in 2013, but implementation has been limited in the face of competing priorities and limited resources. Policy makers highlighted political commitment as a key enabler to address the challenges (Fig. 1). Reinforcing leadership and strengthening the capacity of the health system for prevention and control of NCDs was a key objective in the national policy [34].

### Creating explicit processes for priority setting

The scoping review [31] highlights the evidence gaps relating to NCDs in Sierra Leone – both in relation to risk factors and burden of disease, where evidence is limited, but also in relation to intervention assessment. Only one study focusing on an NCD intervention specific intervention was found: a pilot project aimed at reducing substance abuse [36]. The lack of contextual data and evidence hinders priority setting. Implementation of programmes also tends to be very resource-dependent, implying that external players and donors have a particularly influential role [37]. Within the NCD and mental health directorate, policy priorities include regulatory, fiscal and educational reforms to ensure prevention and promotion in relation to tobacco, alcohol, diet, exercise and road injuries, as well as better management of NCDs at primary care level [38]. The priorities reflect international best practice. However, as noted, policy development and policy implementation are distinct, with the latter considerably more challenging in this fragile and post-conflict context.

### Strengthening interagency cooperation

An interim national technical working group was established in 2018 to coordinate the (relatively few) actors supporting NCD activities. Chaired by the Director of the NCD and Mental Health Directorate at the MoHS, other members represent tertiary medical institutions, development-partner supported programmes, non-governmental organisations and research groups. This interim group is being used on an ad hoc basis, and does not as yet have a strong intersectoral presence from other public bodies with a role to play in relation to addressing the social determinants which drive NCDs. There are plans to set up a substantive Technical Working Group in the very near future that encompasses all relevant actors.

Currently there is limited coordination on NCD activities; in the few districts where this exists, it has been facilitated by external development partner support. NCD coordinating committees are planned in the national policy, but are not yet operational. However, the NCD and mental health directorate is in the process of establishing an NCD and injury commission in line with the Lancet NCDI Poverty Commission [39], with the inaugural meeting already held, to develop policies that address material poverty and integrated health service delivery strategies.

Facility Management Committees (FMC) and Village Development Committees (VDC) (mainly in Bombali District) chaired by the community leaders, play an important role in coordinating the relationships between the CHCs and other stakeholders. The committee organizes monthly meetings, involving stakeholders including chiefs, community health workers (CHWs), drug peddlers, traditional healers, traditional birth attendants, and youth representatives. Our key informants perceived this as a potential platform to conduct NCD prevention and control. For instance, in one CHC, they discussed outreach activities, symptoms of hypertension and diabetes, and conduct education on hypertension and diabetes during the monthly meeting. These stakeholders will then go back to the communities and convey these messages to the residents.

### Enhancing population empowerment

**Fig. 2:**
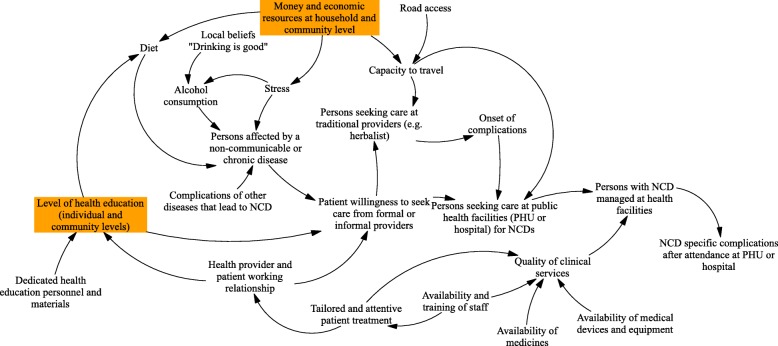
Causal loop diagramme: NCD providers, Bombali. Note: Orange boxes are participant identified points of ‘fragility’ – i.e. areas exhibiting particular weakness within the systems of care

**Fig. 3:**
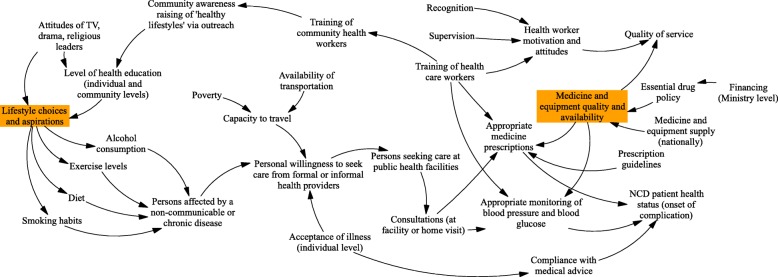
Causal loop diagramme: NCD providers, Western Area. Note: Orange boxes are participant identified points of ‘fragility’ – i.e. areas exhibiting particular weakness within the systems of care

The national NCD policy is built around eight key principles, including ownership and accountability, people-centred health care, cultural relevance, reducing inequities, encompassing the entire care continuum and involving the whole of society, with health promotion and education a priority [34]. However, there are no programmes in operation specifically targeted at health literacy or knowledge of service entitlements in relation to NCDs [31] and generally, providers in our model building workshops reported that community members had relatively high levels of knowledge of risks, but limited awareness of how to address these and limited willingness to do so, given other household priorities. The importance of outreach and health education to address underlying causal factors was highlighted in Western Area and Bombali (Figs. 2 and 3).

In relation to care-seeking, NCD services should be available at health centres, but group model building participants emphasized that quality of care was perceived as poor given medicine stock-outs (which leave patients paying for their own medications, where these are available, or not seeking care at all), and distance (physical and social) to formal health providers. Informal providers are the preferred source of care-seeking for many people. No formal peer-support groups exist, and there are notable differences in access between rural and urban environments, principally due to affordability and distance related barriers, as highlighted in our systems models (Figs. 2 and 3).

According to our key informants, Sierra Leoneans are adopting western lifestyles. Urbanisation has contributed to unhealthier lifestyles. Smoking and alcohol use are very common, while shisha smoking among adolescents and adults has increased recently. Sedentary lifestyles are an important risk factor as community perceptions discourage active physical exercise. Due to these reasons and poor awareness and lifestyles, diabetes and hypertension are perceived as increasing.

Some residents perceive hypertension as a result of ‘too much blood’ in the system, while diabetes is caused by ‘too much sugar’. Patient denial of diseases - not recognising or being willing to accept the disease condition - is common (Fig. 3). Lack of awareness among NCD patients often worsens their disease conditions and delays the access to NCD care. For minor symptoms, people often self-refer to herbalists and traditional medicine vendors, only seeking formal care when complications arise – e.g. with a stroke.

### Establishing effective models of service delivery

Providers have received some training on NCDs during their pre-service training but there have been no national guidelines or desk guides for NCD management at primary care level. This was highlighted by national stakeholders as an important and feasible area for engagement (Fig. 1), and is now being addressed [40]. Service delivery has been characterized as ad hoc, dependent on knowledge of the providers and availability of equipment - both of which are variable across the sites visited. Referral systems for management of chronic disease are weak. Our group models indicated that referral and counter-referrals (for counselling and follow-up, for example for diabetes patients) may operate marginally more effectively in rural areas where community cohesion helps providers identify and trace those persons affected by NCDs.

Limited specialist services are available, mainly within tertiary institutions, e.g. a diabetes clinic is run in Connaught hospital in Freetown. These services often have limited opening hours, which are a further barrier to ensuring effective delivery of care. The national Service Availability and Readiness Assessment (SARA) survey conducted in 2017 reported reasonable levels of NCD service availability, though this does not chime with experience on the ground. 79% of hospitals reported that they offered services for diabetes, 75% for hypertensive disease, 74% for chronic respiratory disease, though only around 40% for cancer care. However, services at primary care level were reported to be much more limited (25% at health centre level for diabetes, 36% for hypertension, 32% for chronic respiratory disease, and around 2% for cancer at all public primary care facilities). Moreover, services were much more commonly available in private than public facilities and in urban than rural ones (by a factor of 3–5 times as common for both categories and across the four disease areas assessed) [41]. The lack of policies to support early detection of NCDs (for example, there is not as yet a national screening programme for cervical cancer, though non-governmental organisations offer this service) can also be a key barrier to accessing care [42]. Access to palliative care remains a problem in low-income countries like Sierra Leone, where the number of deaths from cancer and other NCDs are on the increase [6, 43].

### Establishing coordination across providers

The MoHS has a vision for a coordinated approach to NCDs across levels of the health system, with preventive and outreach work conducted at community level, primary prevention and treatment at primary health units and more acute and complex treatment at secondary and tertiary levels [38]. However, little has yet been implemented. At community level, (CHWs) have recently been established [44], however, their focus is on maternal and child health. Key informants at district level, including CHWs, felt that the CHWs could be a useful resource for NCDs – for example, they could be trained on NCD risk factors and symptoms by the CHOs and could sensitise people on lifestyle risks and encourage residents to come to the community health centres for screening. However, this would require policy change at national level in relation to the roles of CHWs.

Key informant interviews and GMB workshops suggest that referral pathways are not currently effective. Patients often present late with complicated symptoms at secondary and tertiary levels, and discharge planning (down referral) is not usually set in place to provide continuity of care thereafter. Coordination of providers may be marginally better in rural areas, where providers are more connected, our GMBs suggest, but in urban areas, down referral tends to be particularly fragmented.

Across both types of site there is an important presence of private and informal providers, who are not integrated in formal care delivery, posing potential barriers for quality, continuity and loss to follow up. Traditional healers are trusted by many NCD patients, according to key informants, due to their perceived cheaper price and strong belief in traditional treatments by the community [7]. Some CHOs emphasized the importance of establishing good collaboration with traditional healers – for example, encouraging them to identify and refer hypertensive patients.

Others, however, expressed concern about traditional healers overstepping their boundaries. There is intensive cooperation, especially in rural areas, between medical staff and traditional healers of various types. In Makeni, the (DHMT) is trying to integrate traditional healers into the health system by inviting them to attend the DHMT’s monthly meetings. A DHMT officer commented that traditional healers played a cooperative role during the Ebola period, when they helped to spread information to communities. Religious groups, such as churches and mosques, can also play an important role in NCD prevention and care.

### Taking advantage of economies of scale and specialisation

There are a few national centres managing complex NCD care, which entail patients travelling to Freetown [31]. Achieving economies of scale appears to be less the challenge for Sierra Leone than ensuring adequate general coverage of NCD services, with well managed primary care, and responsive referral and counter-referral pathways.

### Creating the right incentive systems

The MoHS plans interventions using taxes, regulatory and legislative approaches, and education/awareness-raising to reduce the burden of NCDs [38]. Fiscal proposals include adding a new 30% excise tax on tobacco, increasing tax on alcohol, and introducing taxes on sugar and salt in foods. More regulatory proposals include banning tobacco and alcohol advertising, banning sales of tobacco to or by minors, regulating their production and accessibility, implementing excise tax stamps on tobacco and alcohol products and enforcing drink-driving and speeding laws. In relation to awareness, mass media messaging on reducing risk factors and promoting healthy diets, breastfeeding and physical activity are planned. However, resourcing to develop and implement these initiatives is currently very limited.

As of June 2015, the average implementation rate of WHO’s Framework Convention on Tobacco Control (FCTC) for Sierra Leone was estimated at 9%, based on the number of indicators implemented for each assessed Article (16 Articles), which led to Sierra Leone ranking the last among the 23 African countries assessed [45]. However, efforts are being put in place, with support from the United Nations Development Programme, World Bank and WHO through the FCTC 2030 project, to improve on this. The NCDs and Mental Health Directorate has recently called on meetings for stakeholders (Parliamentarians, Civil Society Organisations, Religious Leaders, Chiefs, Councillors, Tribal and Community Leaders), to present the tobacco control bill, the investment case report for tobacco control and also educate them on other important tobacco control initiatives the MoHS is proposing. These include Taxation measures, addressing the issue of Illicit Trade and the importance of Earmarking funds.

### Integrating evidence into planning

International evidence has been incorporated in MoHS strategies and plans, however, there is an urgent need, according to GMB stakeholders and key informants, for more locally generated evidence on the prevalence of risk factors, the burden of NCDs, adapted approaches to addressing them and evaluation of NCD interventions in Sierra Leone. The paucity of published literature in the scoping review reinforces this conclusion [31]. The STEP survey conducted in 2009 was incomplete and the MoHS is still seeking support to conduct a full and updated STEP survey. According to the NCD Policy [34], the evaluation of NCD prevention and control was to be under the guidance of the National Steering Committee (this was never set up, but the NCD and Injury Commission being set up will have this oversight function). However, little progress has been made in implementing and therefore evaluating this policy.

### Addressing human resources challenges

Sierra Leone has struggled with human resourcing issues in general, including training sufficient staff with the right skill mix (including general medical specialists), retaining and motivating them, ensuring their equitable and need-sensitive distribution, and providing supportive management and adequate working conditions so that staff can perform adequately [5, 46]. The effects of the civil war and the Ebola outbreak have contributed to these challenges [47]. Given that most NCD care is provided by non-specialists (appropriate for uncomplicated cases), these general constraints affect NCD services. In addition, key informants commented on the frequent moving of staff and failure to develop clear career progression planning, which implies training is often inefficiently deployed.

In addition, many staff are recognized ‘volunteers’, meaning that they are trained but not yet on payroll, working in public facilities at different levels for small stipends or unpaid (this was even true for 35% of doctors, according to the SARA survey [41]. Some of those interviewed attributed their inability to get onto the payroll as being due to poor social connections, which hints at corruption in the recruitment process and is also likely to fuel demands for informal payments from patients.

Work is on-going to improve the basic and in-service training for NCDs (highlighted as a need by national stakeholders – see Fig. 1), including for CHOs, who manage most of the uncomplicated cases and also support outreach and prevention work by community health nurses (and, potentially, CHWs). This needs to be accompanied by roll-out of the newly developed national guidelines for NCD prevention and management, and addressing other system constraints, including lack of essential NCD equipment and medicines (see below) [41]. Further assessments into the competence of managing NCDs by different cadres, perhaps linked to assessing the effective roll-out of new tools, would be useful. This could direct in-service training, which is currently ad hoc and typically project-linked, with little emphasis on NCDs, according to key informants.

Supporting CHOs and other primary care staff with skills related to behaviour change will also be critical. Key informants highlighted the challenge of providing lifestyle counselling to NCD patients, especially when it may not be within the person’s ability to adhere. Health talks are given by community health nurses in the health centres and during outreach activities. However, these talks have previously been mainly focused on maternal and child health, immunization and malaria control, and do not give prominence to the growing NCD threat.

### Improving access to quality medicines for NCDs

Providers across all GMB workshops noted the lack of medicines in public facilities as the single biggest obstacle to delivery of NCD services, driving patients to purchase from private pharmacies or informal sources, with knock-on issues for quality of medicines (see Fig. 3, for example). The SARA report suggested that aspirin, beta blockers, ACE inhibitors, insulin injections, metformin, thiazide, and statins are only available at 29, 5, 4, 3, 5, 5, 2% of health facilities respectively [41]. The lack of national budget line or programme funding for NCD medicines, and general problems with the central purchasing of pharmaceuticals contribute to this problem. While the Free Health Care Initiative [48] has focused on providing free medicines for pregnant and lactating women and under-fives, NCD medicines for all other groups fall within the cost recovery schemes of pharmacies at health centre and hospitals, and the medicines, even when available, may not be affordable to patients and their families. This drives patients to purchase poor quality medicines from informal vendors or forego access completely. Given the chronic nature of NCDs, continuity of access to affordable and effective medicines is essential. Those facilities with access to medicines, such as the private faith-based clinic in Makeni that provides free insulin treatment, face high patient loads. Basic diagnostic equipment is also needed, such as glucometers, glucose strips, blood pressure machines and scales, though the SARA suggested fairly high levels of availability of blood pressure machines (81%) [41].

The government supplies methyldopa, an anti-hypertensive medicine only suitable for high blood pressure in pregnancy, to health centres, but this is not reliable. According to key informants, CHOs therefore self-organise by purchasing and stocking medicine, and selling them on to patients (with payments in cash and in kind, for poorer patients). Prices are reported to be dependent on the financial condition of the patients and the transportation costs to purchase these medicines, though practice varies across CHOs. The CHOs see the provision of medicines as attracting patients and increasing the likelihood of adherence to treatment, however, such practices also create a conflict of interest for staff. Some staff explained that they had therefore stopped stocking medicines and provided prescriptions, which patients filled from pharmacies, then bringing medicines to the staff for checking and guidance.

Informal drug peddlers, locally known as ‘pepper doctors’, are illegal but popular among NCD and other patients. Key informants were concerned about their poor diagnosis skills and sub-standard medicines, which lead to complications for patients and also delay healthcare seeking. Some CHOs have worked with the DHMT and local chiefs to try to control the activities of pepper doctors.

However, the absence of pharmacies creates a market for them, and if controlled in one area, they tend to move to more remote villages, where they are less easily detected.

### Strengthening health system management

The MoHS provides stewardship of the whole health system in Sierra Leone, and within that, the Directorate of NCDs and Mental Health leads on policy development, though with a small team. However, at lower levels (district and below) NCDs are integrated within routine health management structures, with relatively little prominence. The FMCs and VDCs at the community level, as autonomous community organisations facilitated by MoHS, play an important role in supporting the local health management. However, their performance may vary depending on the transport incentives provided for the members to convene in the CHCs.

An interim national Technical Working Group was established in 2018–19 to bring together stakeholders in the field, including the Directorate but also tertiary specialists and international partners and researchers. Larger international donors are however notably absent from this sub-sector. The multi-sectoral committee which was envisaged in the National NCD Policy (to engage partners including non-governmental organisations, faith-based organisation, civil society, communities, media, development partners and the private sector in activities related to NCD prevention and control) [34] has yet to be established.

### Creating adequate information solutions

The national health information system in Sierra Leone suffers from many challenges, including lack of completeness and fragmentation [35]. Specifically for NCDs, there are additional problems, as the recording and follow up at patient level has been limited, lacking systems for managing chronic care.

At the primary health unit level, monthly reports on the numbers of patients with selected NCDs, stratified by sex and child and adult age groups, are sent to the District Health Management Team, where they are collated into monthly summaries for the out-patient morbidity forms by the monitoring and evaluation (M&E) officers and sent to the Directorate of Planning, Policy and Information (DPPI), where data can be obtained by the directorate for NCDs and Mental Health. Monthly reports are also compiled at the secondary and tertiary levels by the monitoring and evaluation officers and sent directly to DPPI [9]. However, limited information on NCDs was available at the MoHS, with very little disaggregation by condition and patient group captured in the health management information system. In relation to cancers, according to our GMBs, a national hospital-based registry has recently been established.

### Overcoming resistance to change

In relation to regulatory, legal and fiscal interventions to address the social determinants of NCDs, relatively limited action has been taken to date – with the exception of a draft Tobacco Bill which is currently ready for review - thus the challenge of overcoming commercial and other interests has not yet occurred.

At the community level, interviews and GMBs highlighted the challenge of changing personal lifestyles and building public awareness of NCDs (Fig. 1). Low levels of health education are highlighted by providers in Bombali (Fig. 2), as well as underlying problems of limited economic resources at the individual and household level (Fig. 2). Poverty and denial of NCD diagnosis were also highlighted in Western Area (Fig. 3). There is also recognition of low trust in formal services by some sections of the community, which erodes health education efforts.

### Ensuring access to care and reducing financial burdens

Physical and financial access to NCD services is a key barrier, highlighted by provider and community GMBs [7] and key informants. Road access was highlighted as an issue in Bombali (Fig. 2), compounded by the long rainy season which makes many roads impassable, while in Western Area, transportation to services was raised by providers.

The lack of funding for NCDs at national level emerged clearly as a blockage (Fig. 1), further influencing the availability of resources at lower levels (Fig. 2). These barriers to service delivery are compounded by poverty from the community side (Fig. 3), implying that overall neither the health system nor community is in a place to address NCDs. The lack of dedicated funding for NCDs, compounded by poor medicines supply, as highlighted above, means that financial barriers remain serious; they are one factor which may be driving families to informal care. Ironically, however, following a chain of treatments through the informal sector can be very costly, as our community GMBs highlighted [7]. There is no policy in place to subsidise care for NCDs, unlike mother and child health care, despite the burden that chronic illness puts on households, with high risk of catastrophic and impoverishing payments. One study reported that 77% of their study population did not seek medical care for NCDs due to lack of financial resources [49].

## Discussion

### Summary of findings

This study has used mixed research methods to populate a framework for assessing health system challenges and opportunities in relation to NCDs in fragile and post-conflict Sierra Leone (Table 3). The results highlight the challenges faced in most domains, which reflect the low priority currently given to NCDs in Sierra Leone as in many other countries in Africa, as well as the overall resource constraints and wider systemic challenges, such as low and poorly distributed health workforce and pharmaceuticals. Findings suggest that health systems opportunities and challenges under most of the domains are similar in both Western Area and Bombali district. However, differences do exist between the two areas. For example, health education at individual and community levels, lack of financial and other resources, and poor road access were highlighted as key constraints in the group model workshops in the rural context of Bombali, though coordination and referral systems were depicted as stronger there, compared to Western Area’s urban context, where medicine and equipment quality, as well as aspirational lifestyle messages were highlighted as key barriers.

**Table 3:** Summary of health system barriers and opportunities for NCD prevention and management in Sierra Leone

Domain of health systems framework	Opportunities	Challenges
Developing political commitment	Reinforcing leadership and strengthening the capacity of the health system for prevention and control of NCDs was a key objective in the national policy.	Efforts have focused on reproductive, maternal and child health, and communicable diseases. Low attention paid until recently to NCD. Implementation has been limited in the face of competing priorities and limited resources.
Creating explicit processes for priority setting	Policy priorities include regulatory, fiscal and educational reforms. The priorities reflect international best practice.	Evidence gaps relating to NCDs mean that the basis for priority setting is absent.
Strengthening interagency cooperation	An interim national technical working group was established in 2018. There are plans to set up a substantive Technical Working Group that encompasses all the relevant actors. The NCD directorate is in the process of establishing an NCD and injury commission. Facility Management Committees and Village Development Committees can be a potential platform to conduct NCD prevention and control.	The interim group does not have a strong intersectoral presence. There is weak NCD coordination at district level where external support is lacking.
Enhancing population empowerment	The national NCD policy is built around eight key principles, with health promotion and education a priority. The importance of outreach and health education to address causal factors is recognised in principle.	No programmes in operation specifically target at health literacy or knowledge of entitlements in relation to NCDs. Knowledge of communities is limited. Perceptions of quality of care is compomised due to medicine stock-outs and distance. Informal providers preferred by some population groups. No formal peer support groups exist. Growing risk factors include: adopting western lifestyles; urbanisation; smoking and alcohol drinking; sedentary lifestyles. Diabetes and hypertension are increasing.
Establishing effective models of service deliver	Providers have received some teaching on NCDs during their pre-service training.	Lack of policies to support early detection of NCDs. Access to palliative care is very limited. No national guidelines or desk guides for NCD management were in operation at primary care level. Care has been dependent on knowledge of the providers and availability of equipment. Public facilities have limited opening hours. Services at primary care level were reported to be limited. Weak referral systems. Limited specialist services are available.
Establishing coordination across provider	MoHS has a vision for a coordinated approach to NCDs across levels of the health system. Community health workers (CHWs) have recently been established. Traditional healers are trusted by many NCD patients. Informal sector can also play an important role.	Little implementation of MoHS policies. Patients often present late with complicated symptoms. Private and informal providers are posing potential barriers for quality, continuity and loss to follow up. Traditional healers reported to be overstepping their boundaries.
Taking advantage of economies of scale and specialisation	Less relevant at this stage given low levels of coverage for NCD services.	
Creating the right incentive system	Fiscal, regulatory and communication proposals developed.	Resourcing is currently very limited and actions have not yet been put in place. Implementation of WHO’s Framework Convention on Tobacco Control (FCTC) for Sierra Leone was estimated at only 9%.
Integrating evidence into planning	International evidence has been incorporated in MoHS strategies and plans.	There is an urgent need for more locally generated evidence. The STEP survey conducted in 2009 was incomplete. The National Steering Committee has not yet been established. Little progress has been made in implementing and therefore evaluating the national NCD policy and strategy.
Addressing human resources challenge	Work is on-going to improve the basic and in-service training for NCDs. Health talks are given by community health nurses in the health centres and during outreach activities.	Human resources for health are generally inadequate in number, distribution and supportive working conditions. Many staff are not yet on payroll. More assessment of competence in managing NCD care by different cadres is required. In-service training is ad hoc and typically project-linked, with little emphasis on NCDs. Staff needs skills-building in a number of NCD-related areas, including providing lifestyle counselling.
Improving access to quality medicines for NCD	The Free Health Care Initiative provides free drugs for pregnant and lactating women and under-fives. The government supplies methyldopa, suitable for high blood pressure in pregnancy to health centres (though this is not reliable). Some CHOs have worked with DHMTs and local chiefs to try to control the activities of pepper doctors.	Lack of medicines in public facilities. Lack of national budget line or programme funding for NCD medicines and general problems with the central purchasing of pharmaceuticals. NCD drugs generally fall within the cost recovery pharmacies at public health facilities. Many purchase poor quality drugs or have no access. A private faith-based clinic in Makeni that provides free insulin treatment faces high patient loads. Basic diagnostic equipment is often lacking or malfunctioning. Staff are in a potential conflict of interest situation if they buy and sell on medicines to patients. Informal drug peddlers are illegal but popular among NCD and other patients.
Strengthening health system management	The NCD and Mental Health Directorate at the MoHS provides overall leadership and NCDs are integrated within routine health system management structures and processes.	NCDs receive relatively little prominence within the sector at all levels.
Creating adequate information solution	Monthly reports sent to DHTM and MoHS.	Health data on NCDs suffers from lack of completeness, fragmentation, and limited detail or disaggregation.
Overcoming resistance to change	A draft Tobacco Bill is currently ready for review.	Relatively limited action has been taken to date. Challenges include low levels of health education, economic resources and trust in formal services.
Ensuring access to care and reducing financial burdens	Some support is available through non-governmental projects and small-scale donor support.	Physical and financial access to NCD services is challenged, especially in rural areas. There is no policy in place to subsidise care for NCDs. Informal sector can be costly and is of unassessed (likely to be low) quality.

### Comparison with wider literature

Our findings fit with the wider picture in in sub-Saharan Africa, where many countries have been slow to develop and implement comprehensive national NCD policies and plans [22, 24]. The challenges documented here are also shared by other fragile settings, which commonly develop national plans and strategies with no real domestic financial or political commitment to support them [22, 24]. NCD control requires improved political will and commitment, raised public awareness to harness resources and increased global attention [50].

At the implementation level, we also found many challenges similar to other sub-Saharan African contexts, such as gaps related to staffing [21], data management system [3], essential drug supply for NCDs in public sector [22], guideline development for NCD management [24], and multi-sectoral collaboration [25]. Our study identified small progress on tobacco control, while previous studies also identify constant interference from the commercial and economic interests of tobacco, alcohol and food industries that continue to drive key NCD risk factors [22, 25, 51].

On the community side, the gap between formal services and their potential users – which a scoping review of fragility and health identifies as one of the key features of fragility [4]– is highlighted here, and will be further explored [7]. Similar to another study in Uganda [23], we identified patients’ poor awareness of NCD treatment and trust in the health care system, which contribute to delays in seeking formal care and to financial burden. Patient financial burdens, together with poor accessibility, equity and responsiveness of primary healthcare services, are widely documented in sub-Saharan Africa literature [26, 27].

### Implications for policy and research

We identify some areas, through the participatory methods, particularly the GMBs, where intervention could be effective, even in this difficult context. In particular, greater national championing could help to drive regulatory and fiscal changes which could be self-financing or even revenue-generating and which could start to address some of the socio-economic factors driving NCDs.

Equally, on the community side, there was enthusiasm for local innovation in introducing behaviour change strategies, working through existing community and district structures. On the supply side, improved training materials for pre- and in-service training for primary care health staff, supported by desk guides and tools for better patient management, were identified and are now being pursued.

Other important ‘key nodes’ such as reliable medicines supply for NCDs are more intractable but research such as this can help promote advocacy, raising the profile of this neglected clinical group, which faces considerable barriers to access and also catastrophic health care (especially medicines) payments.

While our study provides a holistic view of NCD policy implementation in Sierra Leone, there is a need to deepen the understanding of key domains or fragility points in NCD prevention and management. It is also urgent to test interventions which can address structural drivers and change behaviour to reduce risks in these settings but also to improve the service offer, despite limited resources, and develop more effective health care seeking behaviour and financial protection. Based on this study, we have adapted and pilot-tested a package to improve primary care-based hypertension and diabetes management in Bombali district [52] and are exploring social mobilisation and community engagement interventions for NCD prevention in Sierra Leone.

### Reflection on the WHO framework

The framework was developed for the EURO region but this is its first application to a fragile setting, to our knowledge, though different health system assessments have been carried out in low and middle income settings, such as India [53]. The framework was found to be comprehensive enough to capture the key domains of NCD control in this fragile setting. In a context where there are multiple and serious deficits in relation to the various domains, it is important that it is used in a positive manner to identify leverage points and resources, which we have attempted to do, by combining it with a participatory and dynamic methodology, such as the group model building. Future testing of the framework in fragile and conflict-affected settings can help to refine it further.

### Limitations

The methods used have their limitations. They were undertaken in two districts, out of 16 in total, and so are only indicative of the fragile and post-conflict context, though it should be noted that these districts cover about half the population of Sierra Leone and include urban and rural areas. In the absence of current and representative data on NCDs in Sierra Leone, the qualitative methods, which also drew on available literature and secondary data, provided a feasible and relatively rapid method to assess broad barriers and opportunities, which was the objective. More in-depth exploratory and implementation research is needed to understand how to address specific bottlenecks.

## Conclusion

WHO’s NCD assessment framework was tested for the first time in a fragile setting and was found to be helpful in identifying the opportunities and challenges in NCD prevention and management. Our study suggests that NCD prevention and control is of low but increasing priority in Sierra Leone; challenges to addressing this burden relate to huge numbers with NCDs (especially hypertension) requiring care, overall resource constraints and wider systemic issues, including poorly supported primary care services and access barriers. In addition to securing and strengthening political will and commitment and directing more resources and attention towards this area, there is a need for more in-depth exploratory and implementation research to shape and test NCD interventions in fragile and post-conflict states.

## Additional file

**Additional file 1:** Interview topic guides.

**Additional file 2:** Scripts for group model building.

## Data Availability

The datasets used and/or analysed during the current study are available from the corresponding author on reasonable request.

## Abbreviations

CHC: Community Health Centre
CHO: Community Health Officer
CHW: Community Health Worker
DHMT: District Health Management Team
DPPI: Directorate of Planning, Policy and Information
FCTC: Framework Convention on Tobacco Control
FMC: Facility Management Committee
GMB: Group Model Building
LMICs: Low- and middle-income countries
MoHS: Ministry of Health and Sanitation
NCDs: non-communicable diseases
SARA: Service Availability and Readiness Assessment
VDC: Village Development Committee
WHO: World Health Organisation
