# Supplementary material for: Opportunities and challenges for delivering non-communicable disease management and services in fragile and post-conflict settings: perceptions of policy-makers and health providers in Sierra Leone
Source: Confl Health. 2020 Jan 6;14:3. doi: 10.1186/s13031-019-0248-3 (PMC6945746; doi:10.1186/s13031-019-0248-3)
Supplement: Supplementary file 1 — Additional file 1: Interview topic guides. [file 13031_2019_248_MOESM1_ESM.docx]

# Interview topic guides

# National level

Key stakeholders involved in NCD policy formulation and implementation

- Personal profiles(roles, responsibilities, education and career history)
- Roles and responsibilities of the interviewees(e.g., directorate)
- NCD situation in Sierra Leone (SL):
  - Most important health/NCD problems(reminding from scoping review, e.g., obesity, hypertension rates)
  - Why is that?
- Challenges for NCD service delivery
  - Financing: specific funding, perception of patient burden
  - Resources: HR, surveillance system, drugs/supplies/infrastructures,
  - Governance: NCD policy, guidelines, competing priority with infectious diseases?
  - Others
- Current policy priorities and future plans for NCD
- Suggestion and expectation for our research
  - How can we better help?
  - What interventions/areas of improvement should be prioritized?
  - Collaboration between QMU and MoH, and other teams.

# Urban and rural areas

## Primary care level

### Health facility managers

- Personal profiles(roles, responsibilities, education and career history)
- PHUs catchment population, services provided and challenges
- NCD situation in this area:
  - Most important health problems, and impression of the extent of NCD
  - Why is that?(life style, nutrition, post-war/Ebola trauma, stress, etc)
  - Has your facility seen or managed NCD cases? If yes,
    - What is your impression of diagnosing and managing the NCD patients? If possible, give an example?
    - What is your impression of NCD patients and their families (prevention and treatment awareness, financial conditions, etc.)? If possible, give an example?
- Suggestions for interventions/areas of improvement

###

### Health care personnel (diverse set among nurses, community health workers, doctors)

**PHUs (possibly a few CHCs/CHPs/MCHPs)**

- Personal profiles(roles, responsibilities, education and career history)
- PHUs catchment population, services provided
- NCD situation in this area:
  - Most important health problems, and impression of the extent of NCD
  - Why is that?(life style, nutrition, post-war/Ebola trauma, stress, etc)
- Do you receive any training on the NCD training? If yes, content, format and effect.
- Have you seen or managed NCD? If yes,
  - Can you describe or give an example of how you treat and/or manage a NCD patient? [with an aim to understand their care procedures and associated challenges: diagnosis, prescribing, lifestyle education, follow up, adherence, referral, drug supply, etc, probe as necessary)
  - What is your impression of NCD patients and their families (prevention and treatment awareness, financial conditions, etc.)? Can you give an example?
- Suggestions for interventions/areas of improvement

**Community health workers**

- Personal profiles(roles, responsibilities, education and career history)
- Please describe the routine of the community health work? Does your work cover any aspect of NCD service delivery? If yes, how.
- Do you receive any training on the NCD training? If yes, content, format and effect.
- Any challenges of delivering the community health work including NCD/mental health?

## Secondary care level (District Hospitals, possibly in relation to any NCD/MH work)

### Health facility manager

- General information(functions, catchment population, hospital visits, service provided, finance)
- Which departments handle NCD care?
- Main challenges of delivering the NCD service
  - Funding for the hospital, patient cost
  - HR knowledge and skills, surveillance system, drugs/supplies/infrastructures
  - Guidelines, supervision
  - Referral and communication with the PHU
- Suggestions for areas of improvement and intervention

### Health care personnel (diverse set among nurses, doctors, ideally related to NCD/Mental health)

- Personal profiles(roles, responsibilities, education and career history)
- NCD situation in this district:
  - Most important health problems, and impression of the extent of NCD
  - Why is that?(life style, nutrition, post-war/Ebola trauma, stress, etc)
  - NCD visits in your department
- Do you receive any training on the NCD? If yes, content, format and effect.
- Have you seen or managed NCD? If yes,
  - Can you describe or give an example of how you treat and/or manage a NCD patient? [with an aim to understand their care procedures and associated challenges: diagnosis, prescribing, lifestyle education, follow up, adherence, referral, drug supply, etc, probe as necessary)
  - What is your impression of NCD patients and their families (prevention and treatment awareness, financial conditions, etc.)? Can you give an example?
- Suggestions for interventions/areas of improvement

## Tertiary care level

### Health facility manager

- General information(functions, catchment population, hospital visits, service provided, finance)
- Which departments handle NCD care?
- Main challenges of delivering the NCD service
  - Funding for the hospital, patient cost
  - HR knowledge and skills, surveillance system, drugs/supplies/infrastructures
  - Guidelines, supervision
  - Referral and communication with the PHU
- Suggestions for areas of improvement and intervention

###

### Health care personnel (diverse set among nurses, doctors, ideally related to NCD)

- Personal profiles(roles, responsibilities, education and career history)
- NCD situation in this area:
  - Most important health problems, and impression of the extent of NCD
  - Why is that?(life style, nutrition, post-war/Ebola trauma, stress, etc)
  - NCD visits in your department
- Do you receive any training on the NCD? If yes, content, format and effect.
- Have you seen or managed NCD? If yes,
  - Can you describe or give an example of how you treat and/or manage a NCD patient? [with an aim to understand their care procedures and associated challenges: diagnosis, prescribing, lifestyle education, follow up, adherence, referral, drug supply, etc, probe as necessary)
  - What is your impression of NCD patients and their families (prevention and treatment awareness, financial conditions, etc.)? Can you give an example?
- Suggestions for interventions/areas of improvement
