# Supplementary material for: Opportunities and challenges for delivering non-communicable disease management and services in fragile and post-conflict settings: perceptions of policy-makers and health providers in Sierra Leone
Source: Confl Health. 2020 Jan 6;14:3. doi: 10.1186/s13031-019-0248-3 (PMC6945746; doi:10.1186/s13031-019-0248-3)
Supplement: Supplementary file 2 — Additional file 2: Scripts for group model building. [file 13031_2019_248_MOESM2_ESM.docx]

**Scripts for group model building – Sierra Leone**

| **Script 1: Macro level GMB (Policy makers, national actors)** | |
| --- | --- |
| **Time (min)** | **Activity** |
| 10 | Welcome and project introduction |
| 20 | Demonstrating use of GMB methods |
| 30 | **Reference modes**  Participants will be asked to draw graphs over time (last 10 years) depicting the prevalence of NCD conditions (hypertension and diabetes), priority rating these conditions have received over time as well as funding and resource (medicine) availability for these conditions |
| 45-60 | **Identifying points of fragility**  The modelling team presents stakeholders with preliminary concept model structures as elaborated from GMBs conducted at meso/micro and community levels. (10-15 minutes)  Stakeholders are then prompted to identify ‘points of fragility’ within the concept models drawn. (10-15 minutes) A list of potential ‘points of fragility’ will be elaborated and stakeholders will be asked to rank these on an individual basis. (10 minutes)  In plenary feedback, the modelling team will attempt to identify ranking patterns and unveil the set of ‘fragility sources’ elaborated by stakeholders in previous GMBs. Commonalities and differences between the sets will be discussed. |
| 30 | Break |
| 45-60 | **Policy and intervention mapping**  Using the provided concept models, the modelling team will ask stakeholders to draw on/elaborate any key policies or interventions currently planned in relation to NCD care. As relevant, stakeholders will be encouraged to work in groups during this task.  The modelling team will prompt stakeholders to carefully consider the implications of any interventions, including positive and negative spill-over effects, coordination mechanisms as well as implications on resource use. |

| **Script 2: Meso and micro level GMB (health care providers and/or district stakeholders)** | |
| --- | --- |
| **Time (min)** | **Activity** |
| 10 | Welcome and project introduction |
| 10 | Demonstrating use of GMB methods |
| 30 | **Rich pictures**  Ask participants to draw a typical patient with hypertension/diabetes condition (5-10 minutes)  What are the causes for these illnesses/issues that prompt illness development? (5-10 minutes)  Can you draw a journey around these patients? Fill in the drawing with a pre-illness drawing – what were these patients like/doing before/as they arrive to clinic? What is their continued journey likely to be? (10-15 minutes)  Now draw the providers and health care workers surrounding the patient – where are they present? What are their roles? |
| 20 | **Reference modes**  Participants will be asked to draw graphs representing the last 10 years. Graphs ought to focus on: prevalence of hypertension, prevalence of diabetes, knowledge of all conditions among the providers present. |
| 15 | **Break**  Modelling team gathers all materials and elicits/selects a first set of variables to base next activity on. |
| 25 | **Variable elicitation**  Participants are asked to use sticky notes and brainstorm: issues contributing to the onset and exacerbation of each disease, factors affecting the patient journey (from when patient arrives at clinic and then returns home/back to clinic etc), factors affecting a provider’s ability to respond to patient needs (15 minutes)  Comparison of variables (10 minutes): If/where variables coincide, propose a common ‘framing’ of variables be used. Note the diverging variables though allow each group to continue using these as needed going forward. |
| 60-70 | **Causal loop diagram development (Part 1)**  Within each of the groups, the modelling team provides participants with a seed model depicting a patient journey (cascade model: person at risk – person experiencing onset of symptoms) asking them to introduce the variables previously elicited within this.  Prompt the groups to consider where and how the health system – i.e. each of them as providers – impacts on the journey. Start with adding in variables around human resources (e.g. where does the community health worker go? What do they do?) and encourage participants to expand upon the model as much as possible.  Once models are elaborated (approx. 45 minutes), groups will be encouraged to feed back to one another. The possibility of elaborating a single comprehensive model will be explored and facilitators will be interrogating the causal nature of links each time a new link is proposed. |
| 60 | **Lunch** |
| 60-70 | **Causal loop diagram development (Part 2)**  Using models elaborated within the first half of the day, participants will now be asked to work together to merge models – as services are offered within the same spaces and clinics, providers will be encouraged to think of care integration.  Prompt the group to consider issues of resource use, potential guidelines that may come in to assist care delivery, training etc. |
| 45 | **Identifying links or points of ‘fragility’**  The modelling team will prompt participants to identify ‘fragile’ links or points of weakness. Preliminary interviews within Sierra Leone suggest that fragility arises both due to hard- (e.g. limited medicines) and soft-ware (e.g. limited skill training) constraints. The modelling team will encourage participants to identify these within a diagram and potentially rank these using a card sorting task.  Once points of ‘fragility’ are identified, the modelling team will prompt participants to identify various solutions/interventions with potential to improve the situation. |
